# *De novo* clustering of long reads by gene from transcriptomics data

**DOI:** 10.1093/nar/gky834

**Published:** 2018-09-27

**Authors:** Camille Marchet, Lolita Lecompte, Corinne Da Silva, Corinne Cruaud, Jean-Marc Aury, Jacques Nicolas, Pierre Peterlongo

**Affiliations:** 1 Univ Rennes, Inria, CNRS, IRISA, F-35000 Rennes, France; 2 Commissariat à l’Énergie Atomique (CEA), Institut de Biologie François Jacob, Genoscope, 91000 Evry, France

## Abstract

Long-read sequencing currently provides sequences of several thousand base pairs. It is therefore possible to obtain complete transcripts, offering an unprecedented vision of the cellular transcriptome. However the literature lacks tools for *de novo* clustering of such data, in particular for Oxford Nanopore Technologies reads, because of the inherent high error rate compared to short reads. Our goal is to process reads from whole transcriptome sequencing data accurately and without a reference genome in order to reliably group reads coming from the same gene. This *de novo* approach is therefore particularly suitable for non-model species, but can also serve as a useful pre-processing step to improve read mapping. Our contribution both proposes a new algorithm adapted to clustering of reads by gene and a practical and free access tool that allows to scale the complete processing of eukaryotic transcriptomes. We sequenced a mouse RNA sample using the MinION device. This dataset is used to compare our solution to other algorithms used in the context of biological clustering. We demonstrate that it is the best approach for transcriptomics long reads. When a reference is available to enable mapping, we show that it stands as an alternative method that predicts complementary clusters.

## INTRODUCTION

Massively parallel cDNA sequencing by Next Generation Sequencing (NGS) technologies (RNA-seq) has made it possible to take a big step forward in understanding the transcriptome of cells, by providing access to observations as diverse as the measurement of gene expression, the identification of alternative transcript isoforms, or the composition of different RNA populations (1). The main drawback of RNA-seq is that the reads are usually shorter than a full-length RNA transcript. There has been a recent explosion in databases accession records for transcripts obtained with short reads (2) but a laborious curation is needed to filter out false positive reconstructed variants that do not have enough support. Long read sequencing technologies such as Pacific Biosciences (3) and Oxford Nanopore Technologies (4) are referred to as Third Generation Sequencing and make it possible to sequence full-length RNA molecules. In doing so, they remove the need for transcript reconstruction before studying complete RNA transcripts (5). The size of short reads is certainly a major limitation in the process of whole transcript reconstitution, because they may not carry enough information to enable the recovery of the full sequence. In addition, tools for *de novo* assembly of transcripts from short reads (5,6) use heuristic approaches that cannot guarantee the retrieval of exact original transcripts. On the contrary long reads tend to cover full-length cDNA or RNA molecules, and can therefore provide information about the comprehensive exon combinations present in a dataset. This gain in length comes at the cost of a computationally challenging error rate (which varies significantly between protocols, up to over 15%, although RNA reads generally show lower rates, at ∼9% or less (7,8)).

Over the last few years, increasing number of studies have been focusing on the treatment of long read data generated via the Oxford Nanopore MinION, GridION or PromethION platforms, for transcriptome and full-length cDNA analysis (4,9–11). International projects have been launched and the WGS nanopore consortium (https://github.com/nanopore-wgs-consortium/NA12878/blob/master/RNA.md) has for example sequenced the complete human transcriptome using the MinION and GridION nanopores. Besides Human and microbial sequencing, this technology has also proved useful for the *de novo* assembly of a wide variety of species including nematodes (12) and plants (13) or fishes (14). It seems clear that the reduced cost of sequencing and the portable and real-time nature of the equipment compared to the PacBio technology will encourage a wide dissemination of this technology the laboratories (see Schalamun *et al.*, A comprehensive toolkit to enable MinION long-read sequencing in any laboratory, *bioRxiv*, 2018) and many authors point out the world of opportunities offered by nanopores (15). Variant catalogs and expression levels are starting to be extracted from these new resources (16–20), and isoform discovery was cited as a major application of nanopore reads by a recent review (21). However, the vast majority of these works concern species with a reference. In this study we propose supporting the *de novo* analysis of Oxford Nanopore Technologies (ONT) RNA long read sequencing data. We introduce a clustering method that works at the gene level, without the help of a reference. This makes it possible to retrieve the transcripts expressed by a gene, grouped in a cluster. Such clustering may be the basis for a more comprehensive study that aims to describe alternative variants or gene expression patterns.

### Problem statement

**Figure 1.**
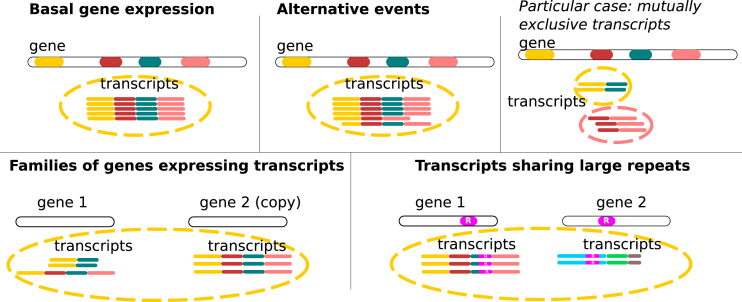
Clustering scenarii. In the case of basal gene expression and alternative events (described below), with the exception of mutually exclusive transcripts, it is expected that all transcripts of a gene will be grouped together in a single cluster. Very small exons or very long retained introns (not shown) can also be limitations according to the mapping tool strategies. In the more complex case of families of genes, two or more copies of paralogous genes can express transcripts at the same time. If these transcripts share a common exonic content and if the gene sequences have not diverged too much (to allow overlap detection), transcripts from this family of genes are clustered together, despite coming from different loci. Although this is an algorithmic limitation, it can be interesting to group these sequences together, as they likely share similar functions. A like scenario occurs for transcripts sharing genomic repeats (such as transposable elements).

Within a long-read dataset, our goal is to identify the associated subset of Third Generation Sequencing reads for each expressed gene without mapping them onto a reference. In order to group RNA transcripts from a given gene using these long and spurious reads, we propose a novel clustering approach. The application context of this paper is non-trivial and specific for at least three reasons: (i) in eukaryotes, it is common that alternative spliced and transcriptional variants with varying exon content (isoforms) occur for a given gene (22). The challenge is to automatically group alternative transcripts in the same cluster (Figure 1); (ii) long reads currently suffer from a high rate of difficult indel errors (7,8); (iii) all genes are not expressed at the same level in the cell (23–25). This leads to a heterogeneous abundance of reads for the different transcripts present. Clusters of different sizes including small ones are expected, which is a hurdle for most algorithms, including the prevalent methods based on community detection (26).

Our method starts from a set of long reads and a graph of similarities between them. It performs an efficient and accurate clustering of the graph nodes to retrieve each group of a gene’s expressed transcripts (detailed in Materials and Methods). A second contribution of our work is an implementation of the clustering algorithm via a tool dubbed CARNAC-LR (**C**lustering coefficient-based **A**cquisition of **RNA C**ommunities in **L**ong **R**eads) inserted into a pipeline (see Results section). The input of this pipeline is a whole dataset of raw reads, with no prior filter or correction needed. The output is a set of clusters that groups reads by gene without the help of a reference genome or transcriptome.

### Background

Early attempts to solve this problem can be traced back to before the age of NGS: in the NCBI UniGene database (27) Expressed Sequence Tags (ESTs) are partitioned into clusters that are very likely to represent distinct genes. In fact, clustering has been the basis for gene indexing in major gene catalogues like UniGene, HGI, STACK or the TIGR Gene Indices (28,29). Moreover this problem has come up in many disciplines, taking different forms according to the application domain. Many studies on sequence clustering worked to find the most efficient way to compute similarity but remained quite basic in their clustering scheme (e.g. CD-HIT (30), SEED (31), Uclust (32), DNACLUST (33)). They essentially used simple schemes to try to avoid all-versus-all pairwise comparison of sequences, which became a major issue with the advent of NGS and meta-transcriptomics. These approaches and the underlying similarity measures were designed for highly similar sequences, and are also popular for applications beyond the scope of this paper such as clustering OTUs. For proteins (34), spectral clustering has been shown to provide meaningful clustering of families. It uses the Blast *E*-value as a raw distance between sequences and takes all of them into account to establish a global partition of protein sequences via simple *K*-means clustering. This type of work cannot easily be extended to the comparison of reads, which are much less structured than protein sequences. To our knowledge no article has been published so far using spectral clustering on RNA reads. For RNA, using Sanger reads then short reads, many approaches used simple single linkage transitive-closure algorithms (EST clustering such as (35–37)), i.e. searched for connected components in a graph of similar sequences. Single linkage clustering is often used for expression data as two similar sequences are meant to merge their clusters into a single one. A problem with simple search for clusters is that it can easily lead to chimeric clusters, especially because of repetitions.

More advanced clustering strategies have therefore been developed for graphs, which use the topological properties of the graph to select relevant classes. Roughly speaking, resolution strategies can be classified into two broad camps according to applications and the community of affiliation: a *graph clustering strategy* based on the search for minimum cuts in these graphs and a *community finding strategy* based on the search for dense subgraphs. Our own approach aims to combine the best of both worlds. The first approach generally searches for a partition into a fixed number of clusters by deleting a minimum number of links that are supposed to be incorrect in the graph. The second approach frequently uses a *modularity* criterion to measure the link density and decide whether overlapping clusters exist, without assumptions regarding the number of clusters. Given that it is difficult to decide on the right number of clusters and to form them solely on the basis of minimizing potentially erroneous links, the main findings and recent developments are based on the community finding strategy and we will focus our review on this approach. *Modularity* measures the difference between the fraction of edges within a single cluster and the fraction of edges that would be observed by chance given the degree of each node. In particular *modularity*-based partitioning of sequences (38) was applied for discovering protein homology (39) or repeat sequence clustering (40). Improved state-of-the-art methods consider either overlapping communities or hierarchical communities. A well-established method for overlapping communities is the Clique Percolation Method (CPM) (41). CPM came with applications such as identification of protein families (42,43).

Finally recent studies (44) rely on the Louvain algorithm (45) that is also based on *modularity* and looks for a hierarchy of clusters, through multi-level optimization that merges the clusters initially reduced to one element as long as *modularity* increases. This algorithm is fast because it uses a greedy strategy and is quite popular for extracting communities from large networks. However, like the other algorithms based on *modularity*, it suffers from two drawbacks: it has difficulty dealing with small clusters and is unstable in that, depending on the order of application of merges, it can produce very different results that are difficult to compare (46). Clustering problems associated with the specifics of long reads start to emerge. Such needs were already a concern in past long read literature (18,47) and are even more acute when a mapping strategy cannot be taken into consideration. We place ourselves in the particular framework of *de novo* identification. While several studies based on long read mapping onto a reference have been produced, methodological contributions that would make it possible to benefit from this promising data remain rare in particular for non model species. To our knowledge, two contributions (47,48) propose respectively *de novo* detection of alternative variants, and clustering and detection of isoforms in long reads transcriptome datasets. However these tools highly rely heavily on the high accuracy provided by Pacific Biosciences Consensus Circular Sequence (CCS) long reads, and therefore do not apply to ONT reads. The method we propose is much more robust to noise.

## MATERIALS AND METHODS

### Input similarity graph

We define a similarity graph as an undirected graph in which nodes are reads and there is an edge between two nodes if the computed similarity between these nodes exceeds a fixed threshold. In such a graph, reads from a single gene are expected to be connected with one another because they are likely to share exons. In the ideal scenario, all reads from a gene are connected with one another. It is therefore a clique. However, the spurious nature of data imposes the use of heuristics to detect read overlaps.

In addition to the presence of genomic repeats, this leads to the expectation of a graph with both missing edges (connection missed during the search for overlapping reads) and spurious edges (wrong connections between unrelated reads), which motivates the development of tailored clustering methods.

### Clustering long reads

#### Clustering issue and sketch of the algorithm

##### Problem formalization

In what follows, we describe the clustering algorithm that is the main contribution of this paper. Our method makes no assumption regarding the number of expressed genes (i.e. clusters/communities), or on the size distribution of such communities, and it needs no input parameter value in the sense that all necessary values are estimated on the data. Since we want to produce a partition of the graph, there are no intersecting communities (no read belongs to more than one gene family) and every node belongs to a community (each read is assigned to a gene). As mentioned previously, the expected subgraph signature of a gene in the graph of reads is a community, that is, a cluster of similar reads. Clustering seeks to maximize intra-cluster similarity and minimize inter-cluster similarity. To measure the density of a connected component, we use the clustering coefficient (*ClCo*) (49) rather than *modularity*. Indeed, we assume that a gene should be represented by a complete subgraph (clique) in a perfect similarity graph. The value of *ClCo* measures the concentration of triangles in a given subgraph (see ‘Selection of community founding node’ section), and this coefficient is more directly connected to the notion of clique than *modularity*. Although we have designed a method that does not require parameter tuning, its foundation depends on two parameters, the number *k* of clusters and the cutoff θ on the *ClCo* value. Specifically, the original problem is formalized as follows:

**Definition 1**. *A community is a connected component in the graph of similarity having a clustering coefficient above a fixed cutoff θ. An optimal clustering in k communities is a minimum k-cut, that is, a partition of the graph nodes into k subsets, that minimizes the total number of edges between two different subsets (the cut-set*).

We assume that the overlap detection procedure (section ‘*First step: computing similarity between long reads*’) has good specificity (it produces a low rate of spurious overlaps). This can be ensured by carefully tuning the parameters of this procedure. The logic behind the search for a minimum cut in the graph is that most of the edges of the initial graph should therefore be kept during clustering. This problem is known to be NP-hard for *k* ≥ 3 (50). Another source of complexity is that we do not know the number of communities in advance, so we have to guess the value of *k*. The *k*-cut should therefore be computed for each possible value between 1 and the maximum, which is the number of reads. Solving this problem is not feasible for the large number of reads that have to be managed. We are therefore looking for an approximation of the solution by using an efficient heuristic approach exploring a restricted space of values for *k*. Finally, the second parameter, the cutoff θ, is not known either. The algorithm thus has to loop over all possible values, that is, all *ClCo* values for a given connected component. In practice it is enough to sample a restricted space of possible *k* values.

##### Algorithm overview

In brief, our community detection algorithm is composed of two main steps. The first one looks for an upper bound of the number of clusters *k*. To this aim, we relax the disjointed communities condition and look initially for star subgraphs (a read connected to all reads similar to it) having a clustering coefficient over a certain cutoff. This corresponds to detecting well-connected reads, called seed reads, using *ClCo* and node degrees (detailed in section ‘*Selection of community founding nodes*’). They form the basis of communities with their neighborhood.

The main challenge is then to refine the boundaries of each community (section‘*Refinement of community boundaries*’) in order to fulfill the partition condition. During this process, the value of *k* is progressively refined by potentially merging clusters whose combination produces a better community (greater *ClCo* value). The other possibility of refinement is to assign nodes to a community and remove them from another. If *x* edges between a node and its previous community are removed, the cut size of the partition is increased by *x*. This core algorithm is run for different cutoff values to obtain different partitions that we compare. We keep the partition associated with the minimal cut (i.e. number of edges removed when computing the partition). The pseudocode of the implemented algorithm is given in ‘Supplementary material’. We set out the different implementation steps in detail below.

#### Generation of partitions

**Figure 2.**
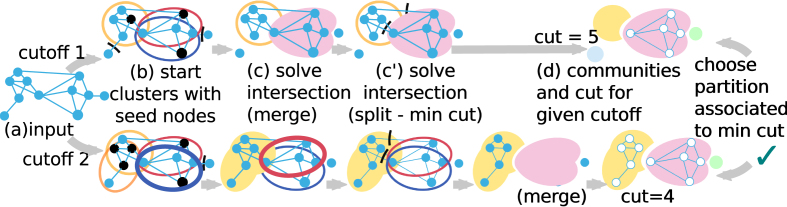
Summary of the algorithm. (**a**) All *ClCo* and degrees are computed. Each *ClCo* value is a cutoff. For a given cutoff, (**b**) different cutoffs yield different seed nodes (black stroke) that initiate clusters with their neighborhood (section ‘*Selection of community founding nodes*’). (**c, c**’) Boundaries of each cluster are then refined. Intersection between clusters are solved either by (c) merging them or by (c’) splitting (section ‘*Refinement of community boundaries*’). (**d**) The communities at different cutoffs evolve in different partitions. In the end we keep only the best partition according to our criterion, i.e. minimizing the cut.

In order to generate and compare different partitions for the graph, we define cutoffs that govern the generation and refinement of communities. The cutoffs can be seen as the level of connectivity at which a community can be generated ((a,b) steps and (c) merge step in Figure 2). In the basic algorithm, for each connected component, all *C*_*l*_*C*_*o*_ are computed in the first place, and partitions are built for each non-zero *ClCo* value as a cutoff. In the end, only one partition is retained, associated with the minimum cut (step (d) in Figure 2). However we have reduced the number of possible cutoff values for the sake of scalability (see ‘Implementation choices for scalability’ section in ‘Supplementary material’). Each step is described for a given cutoff value below.

#### Selection of community founding nodes

Let $\mathcal {G} = (\mathcal {N}, \mathcal {E})$ be an undirected graph of reads. Let *n*_*i*_ be a node (read) from $\mathcal {N}$ and $N_i \subset \mathcal {N}$ its direct neighborhood. Let *deg*(*n*_*i*_) be the number of edges connecting *n*_*i*_ to its direct neighbors (similar reads), i.e. *deg*(*n*_*i*_) = |*N*_*i*_|. For each node *n*_*i*_ ∈ *N* with degree *deg*(*n*_*i*_) > 1, first we compute the *local clustering coefficient*:

(1) \begin{equation*} ClCo_{i} = \frac{2 \ \left|{\lbrace (n_j,n_k) \in \mathcal {E} : n_j,n_k \in N_i\rbrace }\right|}{deg(n_i) \times (deg(n_i) - 1)} \end{equation*}

Nodes of degree 0 and 1 have a *ClCo* of 1. This local coefficient represents the *cliqueness* of the *N*_*i*_∪*n*_*i*_ set of nodes. The closer the coefficient is to 1, the more the set of nodes is inter-connected, which suggests a group a reads that potentially come from the same gene. By contrast, the subgraph induced by a node with a *ClCo* of 0 and its neighbors is a star (i.e. a tree whose leaves are all the neighbours). If the coefficient is close to 0, the nodes are weakly connected and are unlikely to come from the same gene. In order to prevent unwanted star patterns, we add a statistical precaution to prevent star-like patterns (with a very low *ClCo* with respect to the degree of the seed node) from initiating communities. We state the following auxiliary condition for seeds:

(2) \begin{equation*} \forall n_i, ClCo_i \in \: ]cutoff, \theta _2[ \Rightarrow deg(n_i) \le \theta _1 \end{equation*}

θ_1_ and θ_2_ are values such that 1% of the observed degrees are greater than θ_1_ and 1% of the observed *ClCo* are lower than θ_2_ (1st and 99th percentiles). The selected seeds and their direct neighbors form the initial communities. At this point it is possible that two or more communities intersect.

#### Refinement of community boundaries

Community refinement aims at solving the conflicts of intersecting communities. Communities intersect because of spurious connections in the graph due to the creation of edges between unrelated reads in the first step. The intersecting communities are looked up pairwise in order to assign nodes of the intersection to a single community. In fact, two cases have to be differentiated. Either the edges between two communities are considered spurious and these communities must be seen separated (*split*, (c’) step in Figure 2 (the pseudocode for the *split* procedure is also given in ‘Supplementary material’), or the edges have sufficient support and the two communities have to be merged to obtain the full gene expression (*merge*, (c) step in Figure 2). In order to decide between the two, we again use the *cliqueness* notion. This time we introduce an *aggregated clustering coefficient* of the union of two nodes *n*_*i*_ and *n*_*j*_ :

(3) \begin{equation*} ClCo_{ij} = \frac{2 \ \left|{\lbrace (n_k,n_l) \in \mathcal {E} : n_k,n_l \in N_i \cup N_j\rbrace }\right|}{|N_i \cup N_j| \times (|N_i \cup N_j| -1)} \end{equation*}

If the value of *ClCo*_*ij*_ is greater than or equal to the current cutoff, we consider that there is a gain in connectivity when looking at the union of the two communities and they are merged. In the other case, the nodes of the intersection are reported to only one of the two communities. We remove the edges connecting these nodes from one or the other cluster depending on which offers the minimum cut. In case of ties for the cut, the algorithm uses a second criterion. It chooses the cut that maximizes the differences of clustering coefficient values across communities. For two sets $N_1,\: N_{1^{\prime }},\: N_{1} \subseteq N_{1^{\prime }}$, this difference is defined as:

(4) \begin{equation*} \Delta CC_{N_1,N_{1^{\prime }}} = CC_{N_{1^{\prime }}} - CC_{N_{1}}, \end{equation*}

with *CC* calculated as in Equation (1), *N*_1_ being the community before the merge and $N_{1^{\prime }}$ being the community after the merge. The overall result depends on the order in which pairs of clusters are compared. This order is carefully designed. First, the communities associated with the two nodes of greatest degree (and secondly maximum *ClCo*) are chosen, the intersection is resolved and the first community is updated. Then, it is compared to the third best community that intersected if appropriate, and so on until all intersections are solved. This way, we start the comparison with the most promising communities that combine reliability (well-connected subgraphs) with a high potential for resolution (they likely to be the biggest communities, thereby solving intersections for many nodes). On the contrary, communities associated with small subgraphs and relatively low *ClCo* are only resolved afterwards.

#### Complexity and Implementation choices

Our algorithm has a quadratic component in order to compare sets to generate clusters. In addition, it explores the whole space of clustering coefficients with fixed cutoffs. This results in a time complexity that could theoretically be cubic in the number of reads at worst, which is incompatible with processing large datasets.

In order to cope with noise in the input graph, we introduce features to simplify the graph (disconnect loosely connected nodes) and to control the space for looking for possible partitions. In practice these features are also key to reducing the complexity of our approach. Our experiments showed that the running time is reasonable, clustering millions of reads in a few hours. Two key ideas for obtaining this result have been reducing the number of cutoffs and disconnecting the articulation points (51) to reduce the size of connected components in the graph. Details are given in ‘Supplementary material’. Indeed, the most costly phase involves processing of the largest connected components. In these components, many clustering coefficients values are very close and variation in them is mainly an result of noise. Introducing a rounding factor when computing the *ClCo* results in a neat decrease in the number of different values observed, which drastically limits the number of iterations required for the main loop, while providing a very good approximation of the minimum cut. In addition, an upper bound is set on the number of sampled values (100 by default).

We also chose to disconnect the graph’s *articulation points* in order to remove nodes to be targeted as potential bridges between two correct clusters. These are nodes whose removal increases the number of connected components in the graph. Such nodes can be identified as problematic, since we do not expect a single read to be the only link between many others. They can be detected with a DFS for the whole graph in linear time.

Our algorithm has been also carefully designed with respect to the features of long read clustering. To obtain a O(*n*.log(*n*)) complexity with respect to the number *n* of reads, we have made the following assumption: The degree of each node is bounded by a constant, i.e. there is a limited number of transcripts that share similar exons. This ensures that the clustering coefficient of all nodes is calculated in linear time. The most complex operation is the initial sorting of nodes, first by decreasing degree value, then by decreasing *ClCo* value, which can be achieved in O(*n*.log(*n*)). Moreover, since each cluster is initially built on a seed read (see ‘Selection of community founding nodes’ section), it intersects with a bounded number of clusters. Since the loop for making a partition from overlapping clusters is based on a scan of intersections, it is achieved in linear time with respect to the number of reads.

### Validation procedure

#### Production of validation material

##### RNA MinION sequencing

cDNA were prepared from four aliquots (250 ng each) of mouse commercial total RNA (brain, Clontech, Cat# 636601 and 636603), according to the ONT (UK) protocol ‘1D cDNA by ligation (SQK-LSK108)’. The data generated by MinION software (MinKNOWN, Metrichor) was stored and organized using a Hierarchical Data Format. FASTA reads were extracted from MinION HDF files using poretools (52). We obtained 1 256 967 nanopore 1D reads representing around 2GB of data with an average size of 1650 bp and a N50 of 1885 bp.

##### Mapping to obtain reference clusters for validation

We compute ‘ground truth’ clusters for the purposes of validation, using a sensitive third-party protocol based on mapping onto a reference. Nanopore reads from the mouse brain transcriptome were aligned to the masked mouse genome assembly (version GRCm38) using BLAT (53) used for isoform identification with long reads in various studies (21). For each read, the best matches based on the BLAT score (with an identity percent >90%) were selected. Then, those matches were realigned onto the unmasked version of the genome using Est2genome (54) that is dedicated to precise spliced-mapping onto reference genomes. Reads that corresponded to mitochondrial and ribosomal sequences were discarded. Next, nanopore reads were clustered according to their genomic positions: two reads were added to a given cluster if they shared at least 10 nucleotides in their exonic regions. For the whole data experiment, all reads that could be mapped on the reference were taken into account (501 787). Due to repeats (paralogy, transposable elements, etc), some reads were mapped at multiple loci on the reference. When a given read maps on several loci, such loci are gathered into a single expected cluster (12 596 expected clusters). This means that for instance reads from copies of paralog genes that have not diverged to much or reads containing a copy of a transposable element are expected to be in the same cluster.

#### Assessment metrics for cluster accuracy

To assess the results, we used recall and precision measures, which are standard measures for assessing the relevance of biological sequence clustering (55). The recall and precision measures are based on reference clusters obtained by mapping for this validation. They are computed based on representative graphs (56). These measures had already been used to assess the relevance of biological sequence clustering (55). Let $\lbrace \mathcal {C}_1,\ldots \mathcal {C}_i\rbrace _{1 \le i \le L}$ be the set of clusters found by the clustering method tested, where *L* is the number of predicted clusters. Let $\lbrace \mathcal {K}_1,\ldots \mathcal {K}_j\rbrace _{1 \le j \le K}$ be the set of ‘ground truth’ clusters, where *K* is the number of expected clusters. Let *R*_*ij*_ be the number of nodes from $\mathcal {C}_i$ that are in ‘ground truth’ cluster $\mathcal {K}_j$. We compute the recall and the precision such as:

(5) \begin{equation*} {\rm Recall} = \dfrac{\sum \limits _{j=1}^{K}{\rm max}_i(R_{ij})}{\sum \limits _{i=1}^{L}\sum \limits _{j=1}^{K}R_{ij}} \end{equation*}

(6) \begin{equation*} {\rm Precision} = \dfrac{\sum \limits _{i=1}^{L}{\rm max}_j(R_{ij})}{\sum \limits _{i=1}^{L}\sum \limits _{j=1}^{K}R_{ij}} \end{equation*}

Note that the ‘ground truth’ is not really available and that it is estimated from mapping results onto the reference genome. The recall expresses the mean over all clusters of the fraction of relevant reads in a result cluster out of the expected read population of this cluster. It shows to what extent clusters are complete. The precision expresses the mean over all clusters of the fraction of relevant reads among the population of a result cluster. It shows the clusters’ purity. The *F*-measure is a summary measure computed as the weighted harmonic mean between precision and recall. Recall and precision are tailored to express the confidence that can be placed in the method, according to its ability to retrieve information and to be precise. We also assess the closeness of the computed clusters as compared to mapping approaches. Let $\mathcal {X}_0$ be the reference partition (set of clusters obtained by mapping), and $\mathcal {X}$ the partition obtained using a given clustering method. Then *a*_11_ is the number of pairs of nodes that are placed in a single cluster in $\mathcal {X}_0$ and $\mathcal {X}_1$ and *a*_00_ is the number of pairs for which nodes are placed in different clusters both in $\mathcal {X}_0$ and $\mathcal {X}_1$. *a*_10_ (resp. *a*_01_) is the number of pairs of nodes placed in the same cluster in the reference $\mathcal {X}_0$ (resp. $\mathcal {X}$) but in different clusters in $\mathcal {X}$ (resp. $\mathcal {X}_0$). On this basis, a metric such as the Jaccard index shows the match between the reference and computed partitions:

(7) \begin{equation*} J(\mathcal {X}_0,\mathcal {X}) = \frac{a_{11}}{a_{11}+a_{10}+a_{01}} \end{equation*}

The Jaccard index is between 0 and 1. The closer it is to 1, the more the set of clusters computed by a method is close to the ‘ground truth’ set of clusters predicted.

## RESULTS

All experiments were run on Linux distribution with 24 Intel Xeon 2.5 GHz processors, 40 threads and 200GB RAM available. First we present the tool we have developed and made available for large scale long-reads clustering. We demonstrate it performs well on a canonical example on which other clustering approaches were assessed. We compare our approach to well established community detection methods and demonstrate its relevance to long read application. Then we validate our method’s results by comparing them with independent clusters obtained by mapping a real size dataset. In these two parts (sections ‘Comparison to state of the art clustering algorithms’ and ‘Biological relevance’), reads from the mouse brain transcriptome were used in order to access a ‘ground truth’ via a reference. Then we show that our approach offers an alternative to the classical mapping approach even when a reference is available.

### CARNAC-LR, a software for long read clustering

#### Input/Output

We implemented our novel algorithm presented in section ‘Materials and Methods’, integrated into a pipeline called CARNAC-LR. It starts with the computation of long read similarities via a program called Minimap (57) and then produces the clusters. The pipeline’s input is a FASTA file of reads. The output is a text file with one line per cluster, each cluster containing the read indexes. Each read is represented by its index in the original FASTA file during CARNAC-LR computation. Then using indexes, each cluster can easily be converted to a FASTA file where each read’s sequence is retrieved from the original file (a script is provided for this task).

#### First step: computing similarity between long reads

We chose the Minimap tool for its efficiency and its very high level of precision on ONT and PB (58), with regard to other recent methods that can compute similarity or overlaps between long reads despite their error rate (59–62). To generate the similarity graph for CARNAC-LR, Minimap version 0.2 was launched with parameters tuned to improve recall (-Sw2 -L100). It produces a file of read overlaps in .paf format.

#### Second step: clustering

Minimap’s output is converted into a graph of similarity, where each node represents a read and an edge a sequence similarity between two reads above a certain threshold (see (57)). This graph is then passed to CARNAC-LR that retrieves and outputs the gene clusters. CARNAC-LR benefits from parallelization. A thread can be assigned to the treatment of a single connected component, thus many connected component can be computed in parallel. Further results on scalability are provided in ‘Supplementary material’.

### Method validation

#### Input graph

**Figure 3.**
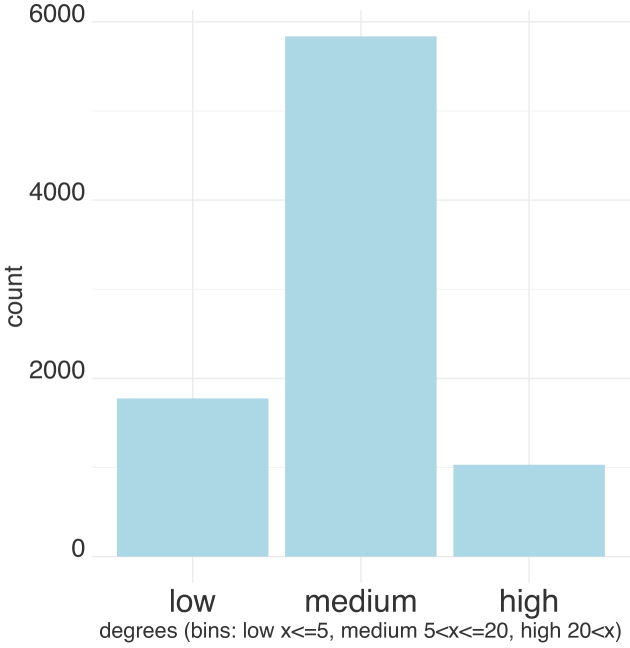
Binned distribution of nodes’ degrees in the input graph.

In order to compare different clustering methods, we generated an input graph from the mouse dataset. We ran all methods on the same input graph, pre-processed using the procedure described in the ‘Complexity and Implementation choices’ section. For scaling purpose, we chose to perform the benchmark on a subset of 10K reads (9609 mouse reads within 527 reference clusters determined by mapping, section ‘*Production of validation material*’). This sampling shows the effect of high gene expression on clustering. We also checked on a second 10K sample from the whole dataset that further accentuates the low expression effect. Directly after Minimap, the graph has 701 connected components, then pre-processing is applied to obtain an input graph that has 1109 connected components. We present a binned distribution of the input graph degrees in Figure 3. Finally, the input graph $\mathcal {G}=\lbrace \mathcal {V,E}\rbrace$ has the following properties: $|\mathcal {V}|=8539$, $|\mathcal {E}|=143317$, graph clustering coefficient: 0.049991, graph average geodesic distance: 8.812268 and graph diameter: 24.

#### Comparison to state of the art clustering algorithms

We show results of state of the art algorithms and compare them to our tool’s results. We compared CARNAC-LR results to algorithms we identified as close to the solution we propose. We evaluated four state-of-the-art methods that have been previously applied to similar biological clustering problems: single linkage transitive-closure (35–37), *modularity* (39,63,64), Clique Percolation Method (42,43) and Louvain (44,65). The results are presented Table 1. Our method has the best precision and the best overall trade-off between precision and recall as shown by the F-measure. It also has the highest Jaccard index among all tested approaches. Louvain’s results were tested for each of its iterations, we present here the best result (other results are given in ‘Supplementary material’). Despite showing the best recall, Louvain’s precision is too low to reach a high F-measure or Jaccard index. The *modularity*-based method achieves average recall and precision values, but one of the lowest Jaccard indices. The transitive closure approach and the CPM are the two other methods that show good results on this instance. The CPM was tested with values for input parameter *k* ranging from 3 to 145 (no community found for greater values). Results are presented for *k* = 3 and show the method’s best performance. For higher values of *k*, the precision increases up to more than 98%, however the recall is dragged down to <15% (details shown in ‘Supplementary material’). Both the CPM and transitive closure present a precision which is >10% inferior to our method. As CARNAC-LR is designed for general pipelines providing a complete analysis of gene variants, it is important that is does not mix two unrelated genes in a single cluster. Our approach is therefore more conservative than CPM and searching for connected components, and it shows comparatively good results in any case. Furthermore it needs no input parameter. Results on the other sample present the same trend as than those presented (shown in ‘Supplementary material’) and demonstrate that CARNAC-LR also deals better with shallow coverage.

**Table 1.** Comparison with state of the art methods. The benchmark was realized on a 10K reads dataset from the mouse chromosome 1. Recall precision and Jaccard Index are presented (see Equations (7), (5) and (6)) to assess for the goodness of communities detection. CPM3 denotes the CPM algorithm using *k* = 3. ‘Clusters’ column shows the number of output clusters of size >1

	Recall (%)	Precision (%)	F-measure (%)	Jaccard index	#clusters
Transitive closure	63.86	87.20	73.72	6.7E−1	731
Modularity	60.70	71.16	65.51	2.6E−1	733
CPM3	63.03	87.17	73.16	6.7E−1	536
Louvain	**81.01**	14.71	28.89	3.6E−2	53
CARNAC-LR	60.16	**98.04**	**74.57**	**7.1 E**−1	748

#### Comparison to other nucleic acid sequence clustering tools

We have simply situated the CARNAC-LR algorithm in relation to existing general cluster detection methods, but we still have to compare our pipeline to other tools dedicated to the comparison of nucleotide sequences that have been developed for the same clustering task. We started with one of the most powerful tools currently available, Starcode (66), which was designed for reads correction and offers a benchmark for the most widely used clustering tools, which we have adopted. This includes CD-HIT (30), SEED (31) and Rainbow (67). It should be noted that none of these tools have been designed to work with ONT reads. They were created before the full development of long reads technology, they have proven not surprisingly completely ill-suited to clustering these long reads. For this test, we used the same mouse dataset as in the previous section. The methods stumble over two features of the data: the error rate and the length of the sequences. SEED for instance is designed to create clusters with sequences that show a maximum of three mismatches, and so finds no clusters. Starcode is not adapted to the size of ONT sequences and terminates with an error message. We tried to increase the maximum size allowed for sequences (initially set at 1024) but the memory consumed continued to grow rapidly and reasonable capacities (200GB) were quickly exceeded. We then tried to perform the calculation by rejecting the longest reads but like SEEDS, Starcode authorizes a limited distance between pairs of sequences (a maximum Levenshtein distance of 8) which is far too small for ONT reads, resulting in singleton clusters. Rainbow only accepts paired reads such as those sequenced in RAD-seq short reads experiments and cannot be adapted to our problem. Finally the most flexible tool, CD-HIT, was the only one to give results. Its ‘EST’ version was used. We tested different values for sequence identity threshold (-c), that can be decreased down to 0.8. We report only the best result, for -c 0.8. It is a long way below the result obtained by CARNAC-LR (*F*-measure up to 41.96% due to low recall, compared with 86.62% for CARNAC-LR). In addition, our pipeline is substantially faster with memory consumption in the same range (within a factor of 2). In view of these results, we added Tofu (48), the only other *de novo* clustering tool that, to our knowledge, is designed to work with long reads, to the benchmark. Unfortunately, Tofu relies heavily on the specificity of Pacific Bioscience RNA protocol (Isoseq) sequences, and cannot be run with ONT reads. Incidentally, the aim of Tofu differs from CARNAC-LR as it is expected to retrieve one cluster per isoform rather than one cluster per expressed gene. A detailed summary of this benchmark result is presented in ‘Supplementary materials’. Once again, another sampling on mouse chromosome 1 was used to perform a second benchmark that presents same conclusions, as also shown in ‘Supplementary material’.

### Biological relevance

#### Validation on a real size dataset

##### Clusters quality

**Figure 4.**
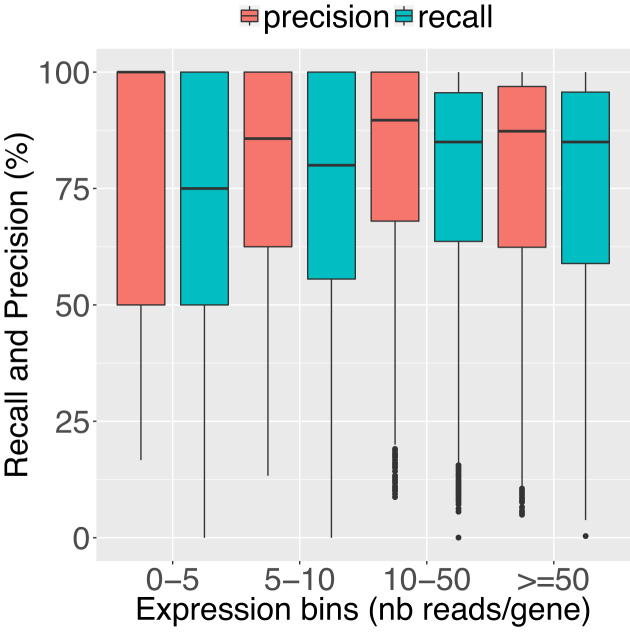
Assessed mean recall and precision of CARNAC-LR+Minimap. They were computed on mouse reads using clusters found by mapping on a reference as a ‘ground truth’ (see Equations 5 and 6). Expression bins are computed from quartiles of expression predicted by mapping and represent the number of mapped reads by gene. Mean precision and recall over all clusters falling in theses bins were then calculated.

In this experiment we demonstrate the quality of *de novo* clusters obtained using CARNAC-LR. We used the subset of reads that could be mapped onto the mouse genome reference (501,787 reads) as a means of comparison for assessing the biological relevance of our clusters. CARNAC-LR’s results were computed using 43 GB RAM and took 18 minutes. The mean recall for CARNAC-LR was 75.38% and the mean precision was 79.62%. In other words, retrieved clusters are on average 75.38% complete, and on average 79.62% of the clusters are composed of unmixed reads from the same gene. In order assess whether our method’s recall and precision is consistent regardless of the gene expression levels, we computed expression bins. For a given gene, we use the number of reads of the ‘ground truth’ cluster to approximate an expression. Any ‘ground truth’ cluster with 5 or less reads is placed in the first bin, and so on for 5–10, 10–50 and ≥50 reads categories. Each of the four bins represent quartiles of expression, which means there is an equal number of clusters in each bin. Figure 4 presents the recalls obtained for binned expression levels and shows our approach’s recall and precision remain consistent despite the heterogeneous coverage in reads. Furthermore, we can deduce from this plot that small clusters do not bias the presented mean recall and precision, as even for big clusters (i.e. ≥50 expression bin) that are more prone to lose some reads, these metrics remain high.

##### Output excerpt

**Figure 5.**
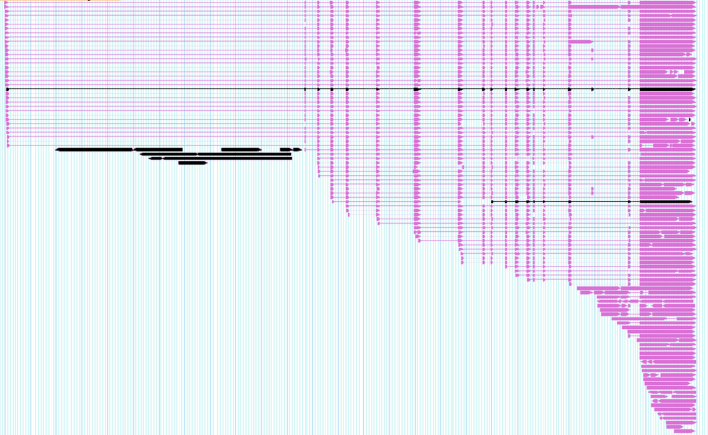
Example of CARNAC-LR’s output cluster in mouse. The output of CARNAC-LR is a text file with one line per cluster, each cluster containing the read indexes. We selected a predicted cluster made of 112 reads (purple). For validation purpose, these reads were mapped with BLAST on an in-house igv (68) version for mouse genome. Reads are spliced-mapped, bold parts are the mapped sequences from the reads and thin parts represents the gaps between the different mapped parts of the reads. Despite the staircase effect observed in the data, this allows to notice that several types of variants were gathered. They could all be assigned to gene Pip5k1c (chr 10:81 293 181–81 319 812), which shows no false positive was present in this cluster. Eight reads (black) present in the data are missed in this cluster. The group of six black reads on the left represent intronic sequences and share no sequence similarity with the others and thus could not appear in the same cluster.

Once CARNAC-LR has been run, FASTA files can extracted for each cluster. We selected the sequences contained in a cluster after CARNAC-LR’s pass on the mouse transcriptome. We used a genome browser to graphically show the reads that were grouped by our approach. (Figure 5). We selected a cluster of sufficient size to be able to present a variety of isoforms. This corresponds to a gene for which mapping retrieved 120 reads. In this example, our approach recovered 93% of the predicted gene’s reads while including no unrelated read in the cluster. Two types of missed reads can be distinguished: (i) A group of black reads that share no genomic sequence with the majority of the gene’s transcript, because they come from an intronic region. These reads could not be linked to the others by Minimap and therefore cannot be clustered with them, as shown in the particular case described in Figures 1 and 2. (ii) Two other reads for which local connectivity was not detected by Minimap were discarded from the cluster. The image shows different exon usage in transcripts, which reveals alternative splicing in this cluster. Different alternative isoforms were therefore gathered in a single cluster as expected (see Figure 1).

#### Complementarity of *de novo* and reference-based approaches

##### Intersection and difference with the set of mapping clusters

Since it does not rely on any reference information, our approach putatively yields different results than classical mapping approaches. In this section, we investigate the differences between the two approaches and demonstrate the usefulness of CARNAC-LR even if a reference is available. We ran it on the full mouse brain transcriptome dataset (1 256 967 reads). We compared the intersections and differences of the results of our approach and mapping. The CARNAC-LR+Minimap pipeline took less than three hours (using 40 threads). In comparison, the ‘ground truth’ clusters took 15 days to be computed (using up to 40 threads). Our approach was able to place 67 422 additional reads that were absent in the mapping procedure, resulting in 39 662 clusters. These clusters fall in two categories (i) *common clusters* with a mix of reads processed by our approach and/or by mapping, or (ii) *novel clusters* that contain reads processed exclusively by our approach or mapping. Each approach performed differently on these categories.

##### Common clusters

For category (i), mapping complemented many common clusters with small amounts of reads left aside by our approach. As some reads are processed by mapping, a recall and precision can still be computed using mapping as ground truth. We computed recall and precision based on the read fraction of clusters that could be compared with mapping. They are quite similar to the values obtained in the previous section (mean recall 75.26% and mean precision 79.30%). This demonstrates that CARNAC-LR efficiently used the supplementary connectivity information despite the addition of potentially noisy reads.

##### Novel clusters

**Figure 6.**
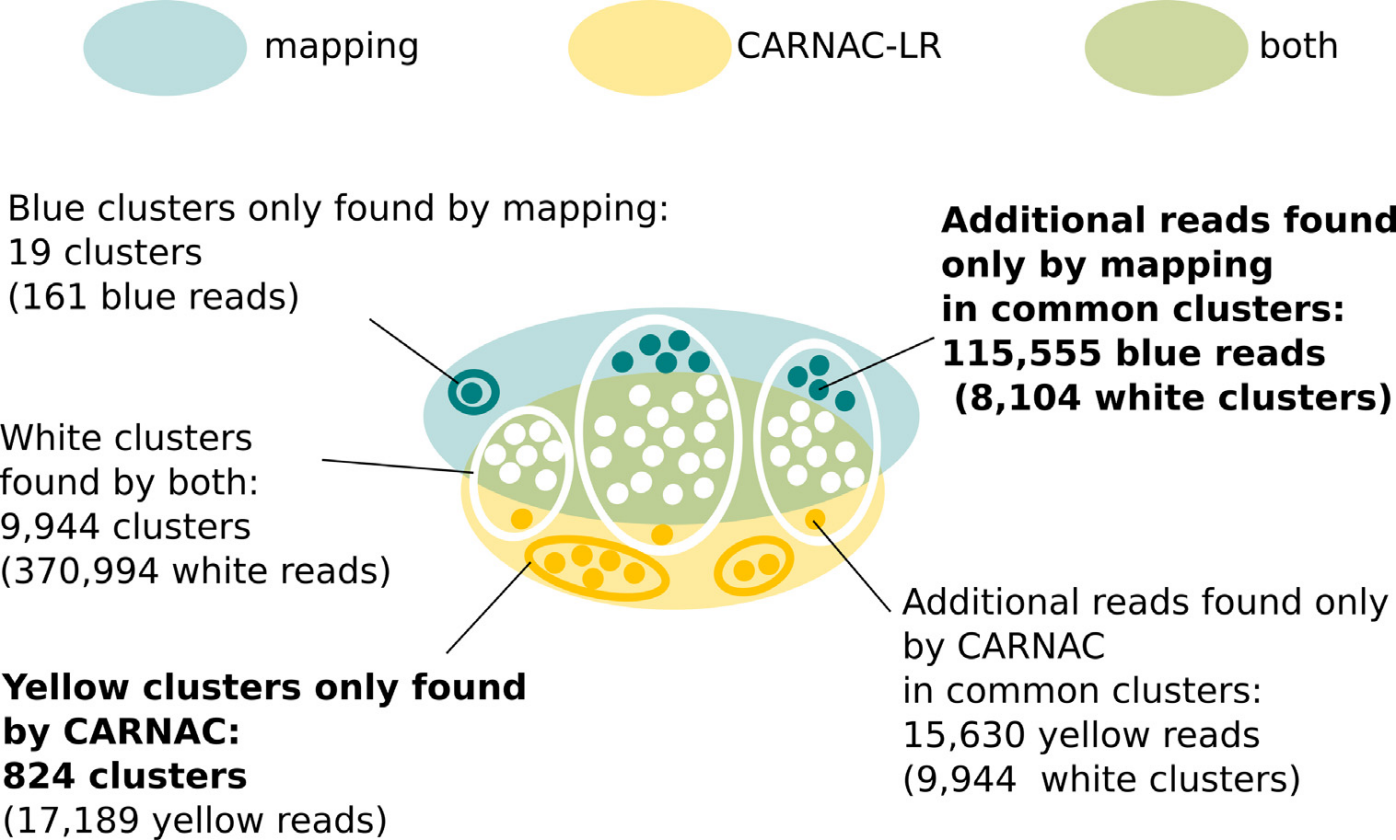
Complementarity of CARNAC-LR and mapping approaches. Only clusters of size ≥5 are represented. Mapping complemented common clusters a with a mean 13 reads per cluster in 90% of clusters. CARNAC-LR’s supply was tenfold lower with a mean 1.3 read added to 100% of common clusters. On the other hand, CARNAC-LR retrieved 15-fold more novel cluster than mapping.

Conversely CARNAC-LR shows a better ability to group reads unprocessed by mapping into novel clusters (Figure 6). CARNAC-LR produced 824 novel clusters (17 189 reads) of category (ii) containing at least five reads. In order to assess the relevance of these novel clusters, we remapped reads *a posteriori*, separately for each cluster, onto the reference genome using a sensible approach (GMAP (69) version 29 September 2015). This operation took ∼10 h (using four threads). 19.68% of mapped reads were assigned to the MT chromosome, then chromosome 11 represented 10.85% of the reads, and other chromosomes <10% of mapped reads each. A third of the reads were multi-mapped (36.7%). However, on average, for each cluster 98.89% of the reads shared a common genomic locus. This is consistent with the expected results of the clustering for reads containing repeats or paralog regions (Figure 1). Finally, 5.7% of the clusters exclusively contained reads mapped at a single locus. All of them could be assigned to an annotated gene. So even if a reference was available, our approach was able to retrieve *de novo* expressed variants of the genes that were unassigned by the mapping.

##### Correlation of expression levels

**Figure 7.**
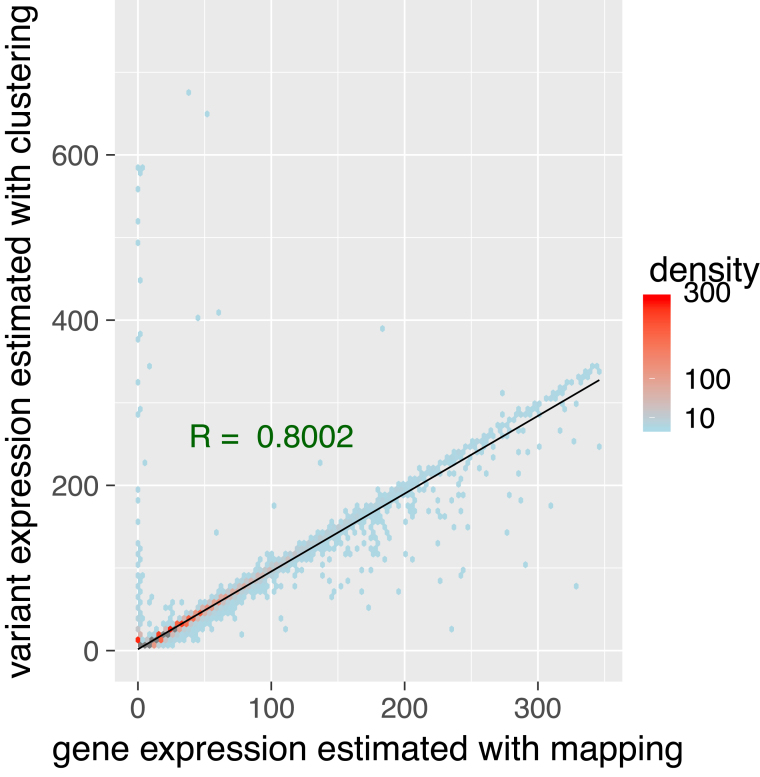
Comparison of clustering and mapping approaches. Comparison and correlation of expressions levels. Gene’s expression can be inferred by counting the number of reads by gene. For each gene, we counted the number of reads retrieved by mapping and we compared it to the number of reads reported by our pipeline and validated by mapping. We computed the Pearson correlation coefficient between the two (in green). Density is the number of points counted in a colored region. Despite a few outliers, we can see a strong linear correlation between the two expression levels estimations (plotted in black). Seven outliers above 750 on Y axis (up to 3327) are not shown.

Another way to look at these results is two consider the number of reads predicted by each method as the gene’s expression, and to compare expression levels predicted by our approach and by mapping. We showed that, despite the previously described differences, they are highly and linearly correlated, with a Pearson correlation coefficient of 0.80 (Figure 7).

## DISCUSSION

### CARNAC-LR is well-suited to transcriptome instances

We demonstrated that our approach can compete with the state-of-the-art algorithms to detect communities. With just a fairly small example, state of the art approaches at least show a lack of precision in comparison to our approach. We showed that a *modularity*-based algorithms such as Louvain algorithm are not well-tailored to this problem, probably because of the heterogeneous size distribution of the clusters, and because of overlapping effects due to the repeats. Among tested state-of-the-art approaches, only the CPM qualifies for retrieving clusters in our input graphs. However, by concentrating its results in a small subset of clusters, it obtains a poor recall and not all its predicted clusters can be trusted. On the other hand our approach shows a good consistency. We supplemented these results with a comparison with tools extensively used for clustering nucleotide sequences, including developments used for EST clustering such as CD-HIT EST. We have shown that no published tool is currently capable of producing quality clusters from ONT RNA reads. We validated CARNAC-LR’s results using mouse transcriptome ONT reads, showing we can compute high confidence clusters for many genes. We underlined that the mapping procedure used for producing reference clusters for validation has its own limitations. Thus the ‘ground truth’ we refer to for the sake of clarity is in fact only partial.

### CARNAC-LR can complement mapping approaches with respect to data with reference

Long reads make it possible to skip the transcript reconstruction step that is necessary with short reads, although this is particularly difficult when it involves assembly. Therefore, long reads constitute an interesting and novel way of obtaining reference transcripts. However, only a fraction of long reads are processed by mappers and downstream analysis is made difficult because of the error rates. In this context, our approach is shown to be an alternative approach to mapping for the identification of gene transcripts. We have shown that our pipeline could be a complementary procedure when reads can be mapped to a reference. It tends to recover some clusters missed by mapping and allows a more efficient use of data. We have demonstrated a straightforward use case for our pipeline as a good proxy for accessing expression levels by gene. ONT sequences have been shown to qualify for transcript quantification in Oikonomopoulos *et al.* (9). In a long read sequencing experiment, it is likely that some reads contain too many errors to be mapped onto a genome. CARNAC-LR can help identifying the origin gene of such reads, if they are clustered with other mapped reads. Moreover CARNAC-LR provides structured information that can be a sound input for other applications. For instance, a read correction step can be performed on each cluster instead of processing all the data, in order to obtain high quality reference transcripts.

### CARNAC-LR applies on non-model species and ONT data

Non model species require *de novo* approaches, and two bioinformatics tools dedicated to them have emerged so far (47,48). Both comprise a pipeline conceived to process Pacific Biosciences Isoseq (3) reads only and require high quality long reads. Thus they could not be used on the data presented here. On the other hand CARNAC-LR is a generic approach that is designed to be used regardless of Third Generation Sequencing error profile and protocol. As a consequence it is the first method to perform *de novo* clustering on RNA reads from ONT.

### Paralogy and repeats

The clustering of sequences from transcriptome reads is made difficult by the existence of multiple repeats. This first attempt to cluster RNA reads by gene is not designed to precisely assign reads from paralog genes to their original locus. We argue that specific instances such as paralog genes constitute research themes on their own and the clustering provides first-approximation results in these cases. We can imagine a second clustering pass with the development of an adapted similarity calculation. CARNAC-LR gathers all reads from a gene family together, provided the different copies have not diverged too much and can therefore be seen as a useful pre-processing step for the analysis of paralogs. A related research axis would be to describe how repeats like transposable elements that can be found in exons or retained introns are treated by the clustering procedure.

## CONCLUSION

We propose a method for clustering long reads obtained from transcriptome sequencing in groups of expressed genes. New algorithmic challenge arises with the combination of a high error rate in the data (7,8), a high heterogeneity of coverage typical of expression data and a large volume of data. In this, our issue differs from EST clustering problems for instance. We demonstrated our method’s relevance for this application, in comparison to literature approaches. It takes reads early after their generation, without correction or filtering. The expressed variants of each gene are obtained from the clusters, and related transcripts are identified, even when no reference is available. To make our solution practical for users, we provide an implementation called CARNAC-LR that, combined with Minimap, scales and is able to quickly process real data instances, as demonstrated by the processing of the whole mouse brain transcriptome.

As a result of the quick development of TGS, the sequencing field is frequently upgraded with new types of sequences. For instance, recent long read technology ONT RNA-direct could unlock amplification biases issues in RNA sequencing and is therefore promising for gene expression studies (see Garalde *et al.*, Highly parallel direct RNA sequencing on an array of nanopores, *bioRxiv*, 2016). But it shows higher error rates, at least compared to reads presented in this study, according to unpublished works. By proposing a tool tailored to ONT, we wish to promote and encourage a broader use of these long reads for transcriptome analysis.

## DATA AVAILABILITY AND IMPLEMENTATION

CARNAC-LR is written in C++, open source and available for Linux systems at github.com/kamimrcht/CARNAC-LR under the Affero GPL license. The nanopore reads from the mouse RNA sample are available from the ENA repository under the following study : ERP107503.

## Supplementary Material

Supplementary Data

## References

[1] Kukurba K.R., Montgomery S.B. RNA sequencing and analysis. Cold Spring Harbor protocols. 2015; 2015:951–969. 25870306 10.1101/pdb.top084970 PMC4863231

[2] O’Leary N.A., Wright M.W., Brister J.R., Ciufo S., Haddad D., McVeigh R., Rajput B., Robbertse B., Smith-White B., Ako-Adjei D. Reference sequence (RefSeq) database at NCBI: current status, taxonomic expansion, and functional annotation. Nucleic Acids Res. 2015; 44:D733–D745. 26553804 10.1093/nar/gkv1189 PMC4702849

[3] Gonzalez-Garay M.L. Introduction to isoform sequencing using pacific biosciences technology (Iso-Seq). Introduction to Isoform Sequencing Using Pacific Biosciences Technology (iso-seq) In Transcriptomics and Gene Regulation. 2016; Springer 141–160.

[4] Weirather J.L., deCesare M., Wang Y., Piazza P., Sebastiano V., Wang X.-J., Buck D., Au K.F. Comprehensive comparison of Pacific Biosciences and Oxford Nanopore Technologies and their applications to transcriptome analysis [version 2; referees: 2 approved]. F1000Research. 2017; 6:100. 28868132 10.12688/f1000research.10571.1 PMC5553090

[5] Grabherr M.G., Haas B.J., Yassour M., Levin J.Z., Thompson D.A., Amit I., Adiconis X., Fan L., Raychowdhury R., Zeng Q. Trinity: reconstructing a full-length transcriptome without a genome from RNA-Seq data. Nat. Biotechnol. 2011; 29:644. 21572440 10.1038/nbt.1883 PMC3571712

[6] Trapnell C., Roberts A., Goff L., Pertea G., Kim D., Kelley D.R., Pimentel H., Salzberg S.L., Rinn J.L., Pachter L. Differential gene and transcript expression analysis of RNA-seq experiments with TopHat and Cufflinks. Nat. Protoc. 2012; 7:562–578. 22383036 10.1038/nprot.2012.016 PMC3334321

[7] Laehnemann D., Borkhardt A., McHardy A.C. Denoising DNA deep sequencing data – high-throughput sequencing errors and their correction. Briefings in bioinformatics. 2015; 17:154–179. 26026159 10.1093/bib/bbv029 PMC4719071

[8] Ip C.L.C., Loose M., Tyson J.R., deCesare M., Brown B.L., Jain M., Leggett R.M., Eccles D.A., Zalunin V., Urban J.M. MinION analysis and reference consortium: phase 1 data release and analysis [version 1; referees: 2 approved]. F1000Research. 2015; 4:1075. 26834992 10.12688/f1000research.7201.1 PMC4722697

[9] Oikonomopoulos S., Wang Y.C., Djambazian H., Badescu D., Ragoussis J. Benchmarking of the Oxford Nanopore MinION sequencing for quantitative and qualitative assessment of cDNA populations. Scientific Rep. 2016; 6:31602. 10.1038/srep31602 PMC4995519 27554526

[10] Bolisetty M.T., Rajadinakaran G., Graveley B.R. Determining exon connectivity in complex mRNAs by nanopore sequencing. Genome Biol. 2015; 16:204. 26420219 10.1186/s13059-015-0777-z PMC4588896

[11] Hargreaves A.D., Mulley J.F. Assessing the utility of the Oxford Nanopore MinION for snake venom gland cDNA sequencing. PeerJ. 2015; 3:e1441. 26623194 10.7717/peerj.1441 PMC4662598

[12] Eccles D., Chandler J., Camberis M., Henrissat B., Koren S., Le Gros G., Ewbank J.J. (1) De novo assembly of the complex genome of Nippostrongylus brasiliensis using MinION long reads. BMC Biol. 2018; 16:6. 29325570 10.1186/s12915-017-0473-4 PMC5765664

[13] Schmidt M.H.-W., Vogel A., Denton A.K., Istace B., Wormit A., van deGeest H., Bolger M.E., Alseekh S., Maß J., Pfaff C. Rapid de novo assembly of the European eel genome from nanopore sequencing reads. Plant Cell. 2017; 29:2336–2348. 29025960 10.1105/tpc.17.00521 PMC5774570

[14] Jansen H.J., Liem M., Jong-Raadsen S.A., Dufour S., Weltzien F.-A., Swinkels W., Koelewijn A., Palstra A.P., Pelster B., Spaink H.P. Rapid de novo assembly of the European eel genome from nanopore sequencing reads. Scientific Rep. 2017; 7:7213. 10.1038/s41598-017-07650-6 PMC5543108 28775309

[15] Leggett R.M., Clark M.D. De novo assembly of the complex genome of Nippostrongylus brasiliensis using MinION long reads. J. Exp. Bot. 2017; 68:5419–5429.28992056

[16] Au K.F., Sebastiano V., Afshar P.T., Durruthy J.D., Lee L., Williams B.A., vanBakel H., Schadt E.E., Reijo-Pera R.A., Underwood J.G. Characterization of the human ESC transcriptome by hybrid sequencing. Proc. Natl. Acad. Sci. U.S.A. 2013; 110:E4821–E4830. 24282307 10.1073/pnas.1320101110 PMC3864310

[17] Sharon D., Tilgner H., Grubert F., Snyder M. A single-molecule long-read survey of the human transcriptome. Nat. Biotechnol. 2013; 31:1009–1014. 24108091 10.1038/nbt.2705 PMC4075632

[18] Abdel-Ghany S.E., Hamilton M., Jacobi J.L., Ngam P., Devitt N., Schilkey F., Ben-Hur A., Reddy A.S. A survey of the sorghum transcriptome using single-molecule long reads. Nat. Commun. 2016; 7:11706. 27339290 10.1038/ncomms11706 PMC4931028

[19] Wang B., Tseng E., Regulski M., Clark T.A., Hon T., Jiao Y., Lu Z., Olson A., Stein J.C., Ware D. Unveiling the complexity of the maize transcriptome by single-molecule long-read sequencing. Nat. Commun. 2016; 7:11708. 27339440 10.1038/ncomms11708 PMC4931018

[20] Hoang N.V., Furtado A., Mason P.J., Marquardt A., Kasirajan L., Thirugnanasambandam P.P., Botha F.C., Henry R.J. A survey of the complex transcriptome from the highly polyploid sugarcane genome using full-length isoform sequencing and de novo assembly from short read sequencing. BMC Genomics. 2017; 18:395. 28532419 10.1186/s12864-017-3757-8 PMC5440902

[21] Sedlazeck F.J., Lee H., Darby C.A., Schatz M.C. Piercing the dark matter: bioinformatics of long-range sequencing and mapping. Nat. Rev. Genetics. 2018; 16:204. 10.1038/s41576-018-0003-4 29599501

[22] Modrek B., Lee C. A genomic view of alternative splicing. Nat. Genet. 2002; 30:13–19. 11753382 10.1038/ng0102-13

[23] Holter N.S., Mitra M., Maritan A., Cieplak M., Banavar J.R., Fedoroff N.V. Fundamental patterns underlying gene expression profiles: simplicity from complexity. Proc. Natl. Acad. Sci. U.S.A. 2000; 97:8409–8414. 10890920 10.1073/pnas.150242097 PMC26961

[24] Rodwell G.E., Sonu R., Zahn J.M., Lund J., Wilhelmy J., Wang L., Xiao W., Mindrinos M., Crane E., Segal E. A transcriptional profile of aging in the human kidney. PLoS Biol. 2004; 2:e427. 15562319 10.1371/journal.pbio.0020427 PMC532391

[25] Schadt E.E., Monks S.A., Drake T.A., Lusis A.J., Chek N., Colinayok V., Ruff T.G., Milligan S.B., Lamb J.R., Cavet G. Genetics of gene expression surveyed in maize, mouse and man. Nature. 2003; 422:297. 12646919 10.1038/nature01434

[26] Fortunato S. Community detection in graphs. Phys. Rep. 2010; 486:75–174.

[27] Schuler G.D. Pieces of the puzzle: expressed sequence tags and the catalog of human genes. J. Mol. Med. 1997; 75:694–698. 9382993 10.1007/s001090050155

[28] Bouck J., Yu W., Gibbs R., Worley K. Comparison of gene indexing databases. Trends Genet. 1999; 15:159–162. 10203827 10.1016/s0168-9525(99)01709-6

[29] Quackenbush J., Liang F., Holt I., Pertea G., Upton J. Resolution limit in community detection. Nucleic Acids Res. 2000; 28:141–145. 10592205 10.1093/nar/28.1.141 PMC102391

[30] Li W., Godzik A. Cd-hit: a fast program for clustering and comparing large sets of protein or nucleotide sequences. Bioinformatics. 2006; 22:1658–1659. 16731699 10.1093/bioinformatics/btl158

[31] Bao E., Jiang T., Kaloshian I., Girke T. SEED: efficient clustering of next-generation sequences. Bioinformatics. 2011; 27:2502–2509. 21810899 10.1093/bioinformatics/btr447 PMC3167058

[32] Edgar R.C. Search and clustering orders of magnitude faster than BLAST. Bioinformatics. 2010; 26:2460–2461. 20709691 10.1093/bioinformatics/btq461

[33] Ghodsi M., Liu B., Pop M. DNACLUST: accurate and efficient clustering of phylogenetic marker genes. BMC Bioinformatics. 2011; 12:271. 21718538 10.1186/1471-2105-12-271 PMC3213679

[34] Paccanaro A., Casbon J.A., Saqi M.A.S. A comprehensive toolkit to enable MinION long-read sequencing in any laboratory. Nucleic Acids Res. 2006; 34:1571–1580. 16547200 10.1093/nar/gkj515 PMC1409676

[35] Dost B., Wu C., Su A., Bafna V. TCLUST: a fast method for clustering genome-scale expression data. IEEE/ACM Trans. Comput. Biol. Bioinformatics (TCBB). 2011; 8:808–818. 10.1109/TCBB.2010.34 20479508

[36] Burke J., Davison D., Hide W. d2_cluster: a validated method for clustering EST and full-length cDNA sequences. Genome Res. 1999; 9:1135–1142. 10568753 10.1101/gr.9.11.1135 PMC310833

[37] Christoffels A., Gelder A.V., Greyling G., Miller R., Hide T., Hide W. STACK: sequence tag alignment and consensus knowledgebase. Nucleic Acids Res. 2001; 29:234–238. 11125101 10.1093/nar/29.1.234 PMC29830

[38] Girvan M., Newman M.E. Community structure in social and biological networks. Proc. Natl. Acad. Sci. U.S.A. 2002; 99:7821–7826. 12060727 10.1073/pnas.122653799 PMC122977

[39] Mei J., Zhao J., Yang X., Zhou W. Remote protein homology detection using a modularity-based approach. Information Science and Technology (ICIST), 2011 International Conference on IEEE. 2011; 1287–1291.

[40] Novák P., Neumann P., Macas J. Graph-based clustering and characterization of repetitive sequences in next-generation sequencing data. BMC Bioinformatics. 2010; 11:378. 20633259 10.1186/1471-2105-11-378 PMC2912890

[41] Palla G., Barabási A., Vicsek T. Quantifying social group evolution. Nature. 2007; 446:664–667. 17410175 10.1038/nature05670

[42] Jonsson P.F., Cavanna T., Zicha D., Bates P.A. Cluster analysis of networks generated through homology: automatic identification of important protein communities involved in cancer metastasis. BMC Bioinformatics. 2006; 7:2. 16398927 10.1186/1471-2105-7-2 PMC1363365

[43] Adamcsek B., Palla G., Farkas I.J., Derényi I., Vicsek T. CFinder: locating cliques and overlapping modules in biological networks. Bioinformatics. 2006; 22:1021–1023. 16473872 10.1093/bioinformatics/btl039

[44] Forster D., Bittner L., Karkar S., Dunthorn M., Romac S., Audic S., Lopez P., Stoeck T., Bapteste E. Testing ecological theories with sequence similarity networks: marine ciliates exhibit similar geographic dispersal patterns as multicellular organisms. BMC Biol. 2015; 13:16. 25762112 10.1186/s12915-015-0125-5 PMC4381497

[45] Blondel V.D., Guillaume J.-L., Lambiotte R., Lefebvre E. Fast unfolding of communities in large networks. J. Stat. Mech.: Theory Exp. 2008; 2008:P10008.

[46] Good B.H., deMontjoye Y.-A., Clauset A. Performance of modularity maximization in practical contexts. Phys. Rev. E. 2010; 81:046106. 10.1103/PhysRevE.81.046106 20481785

[47] Liu X., Mei W., Soltis P.S., Soltis D.E., Barbazuk W.B. Detecting alternatively spliced transcript isoforms from single-molecule long-read sequences without a reference genome. Mol. Ecol. Resources. 2017; 17:1243–1256. 10.1111/1755-0998.12670 28316149

[48] Gordon S.P., Tseng E., Salamov A., Zhang J., Meng X., Zhao Z., Kang D., Underwood J., Grigoriev I.V., Figueroa M. Widespread polycistronic transcripts in fungi revealed by single-molecule mRNA sequencing. PLoS One. 2015; 10:e0132628. 26177194 10.1371/journal.pone.0132628 PMC4503453

[49] Newman M.E. The structure and function of complex networks. SIAM Rev. 2003; 45:167–256.

[50] Dahlhaus E., Johnson D.S., Papadimitriou C.H., Seymour P.D., Yannakakis M. The complexity of multiterminal cuts. SIAM J. Comput. 1994; 23:864–894.

[51] Hopcroft J., Tarjan R. Algorithm 447: efficient algorithms for graph manipulation. Commun. ACM. 1973; 16:372–378.

[52] Loman N.J., Quinlan A.R. Poretools: a toolkit for analyzing nanopore sequence data. Bioinformatics. 2014; 30:3399–3401. 25143291 10.1093/bioinformatics/btu555 PMC4296151

[53] Kent W.J. BLAT – the BLAST-like alignment tool. Genome Res. 2002; 12:656–664. 11932250 10.1101/gr.229202 PMC187518

[54] Mott R. EST_GENOME: a program to align spliced DNA sequences to unspliced genomic DNA. Bioinformatics. 1997; 13:477–478. 10.1093/bioinformatics/13.4.477 9283765

[55] Wang Y., Leung H.C., Yiu S., Chin F.Y. d2_cluster: a validated method for clustering EST and full-length cDNA sequences. Bioinformatics. 2012; 28:i356–i362. 22962452 10.1093/bioinformatics/bts397 PMC3436824

[56] Senior J.K. Partitions and their representative graphs. Am. J. Math. 1951; 73:663–689.

[57] Li H. Minimap and miniasm: fast mapping and de novo assembly for noisy long sequences. Bioinformatics. 2016; 32:2103–2110. 27153593 10.1093/bioinformatics/btw152 PMC4937194

[58] Chu J., Mohamadi H., Warren R.L., Yang C., Birol I. Innovations and challenges in detecting long read overlaps: an evaluation of the state-of-the-art. Bioinformatics. 2016; 33:1261–1270. 10.1093/bioinformatics/btw811 PMC5408847 28003261

[59] Myers G. Efficient local alignment discovery amongst noisy long reads. International Workshop on Algorithms in Bioinformatics Springer. 2014; 52–67.

[60] Berlin K., Koren S., Chin C.-S., Drake J.P., Landolin J.M., Phillippy A.M. Assembling large genomes with single-molecule sequencing and locality-sensitive hashing. Nat. Biotechnol. 2015; 33:623–630. 26006009 10.1038/nbt.3238

[61] Sović I., Šikić M., Wilm A., Fenlon S.N., Chen S., Nagarajan N. Fast and sensitive mapping of nanopore sequencing reads with GraphMap. Nat. Commun. 2016; 7:11307. 27079541 10.1038/ncomms11307 PMC4835549

[62] Chaisson M.J., Tesler G. Mapping single molecule sequencing reads using basic local alignment with successive refinement (BLASR): application and theory. BMC Bioinformatics. 2012; 13:238. 22988817 10.1186/1471-2105-13-238 PMC3572422

[63] Wei Z.-G., Zhang S.-W., Zhang Y.-Z. DMclust, a density-based modularity method for accurate OTU picking of 16S rRNA sequences. Mol.Informatics. 2017; 36:1600059. 10.1002/minf.201600059 28586119

[64] Novák P., Neumann P., Macas J. Graph-based clustering and characterization of repetitive sequences in next-generation sequencing data. BMC bioinformatics. 2010; 11:378. 20633259 10.1186/1471-2105-11-378 PMC2912890

[65] Miele V., Penel S., Daubin V., Picard F., Kahn D., Duret L. High-quality sequence clustering guided by network topology and multiple alignment likelihood. Bioinformatics. 2012; 28:1078–1085. 22368255 10.1093/bioinformatics/bts098

[66] Zorita E., Cusco P., Filion G.J. Starcode: sequence clustering based on all-pairs search. Bioinformatics. 2015; 31:1913–1919. 25638815 10.1093/bioinformatics/btv053 PMC4765884

[67] Chong Z., Ruan J., Wu C.-I. Rainbow: an integrated tool for efficient clustering and assembling RAD-seq reads. Bioinformatics. 2012; 28:2732–2737. 22942077 10.1093/bioinformatics/bts482

[68] Robinson J.T., Thorvaldsdóttir H., Winckler W., Guttman M., Lander E.S., Getz G., Mesirov J.P. Integrative genomics viewer. Nat. Biotechnol. 2011; 29:24. 21221095 10.1038/nbt.1754 PMC3346182

[69] Wu T.D., Watanabe C.K. GMAP: a genomic mapping and alignment program for mRNA and EST sequences. Bioinformatics. 2005; 21:1859–1875. 15728110 10.1093/bioinformatics/bti310

