# Supplementary material for: De novo clustering of long reads by gene from transcriptomics data
Source: Nucleic Acids Res. 2018 Sep 27;47(1):e2. doi: 10.1093/nar/gky834 (PMC6326815; doi:10.1093/nar/gky834)
Supplement: Supplementary Data [file gky834_supplemental_files.pdf]

Supplementary material

Detailed algorithms

Algorithm: Main

Data: Graph $\mathcal{G} = (\mathcal{N}, \mathcal{E})$

Result: A cutoff CC_{min}, and a partition P of $\mathcal{N}$
$P = \{\mathcal{C}(n_1), \mathcal{C}(n_2), ...\mathcal{C}(n_k)\}$ such that
all $\mathcal{C}(n_i)$ have a clustering coefficient $\geq CC_{min}$,
and result in a minimal cut of $\mathcal{G}$ with value Cut_{min}

foreach *node n of* $\mathcal{N}$ **do**
  Compute its degree $deg(n)$;
  Compute its clustering coefficient $CC(n)$ (equ. (1) in main document);
$\mathcal{N} \leftarrow \mathcal{N} \setminus \{n \mid CC(n) = 0$ or is an articulation node in $\mathcal{G}\}$
$S\mathcal{N} \leftarrow sort\_deg(\mathcal{N})$; *% Sorted list of nodes*
$CC_{min} = 0$; $Cut_{min} = \infty$
% Loop over possible cutoff values
foreach *cutoff sampled in CC* **do**
  % Compute possibly overlapping clusters
  foreach n *in* $S\mathcal{N}$ *such that* $CC(n) \geq cutoff$ **do**
    $\mathcal{C}(n) \leftarrow \{n\} \cup neighbours(n)$; *% Initial clusters*
    $S_{cl}(n) \leftarrow$ Sorted list of clusters containing n in decreasing value of CC
    $\mathcal{N}_{Inter} \leftarrow$ Nodes in the intersection of 2 clusters
    $\mathcal{N}_{cl} \leftarrow sort\_degc(\mathcal{N}_{Inter})$

  % Make a partition from overlapping clusters
  foreach *node n in* $\mathcal{N}_{cl}$ **do**
    $x = S_{cl}(n)[1]$ *% Representative element of the cluster*
    foreach y *in* $S_{cl}(n)[2 : length(S_{cl}(n))]$ **do**
      $CC_{xy} \leftarrow$clustering coefficient of $x \cup y$ (equ. (2) in main document);
      if $CC_{xy} \geq cutoff$ **then**
        $x \leftarrow$ **Merge**(x, y); *%* $\mathcal{C}(x) = \mathcal{C}(x) \cup \mathcal{C}(y)$
      else
        $x \leftarrow$ **Split**$(x, y, \mathcal{E})$ (algo[2])
        % n is discarded from one of the clusters
        % x refers now to the cluster containing n
  Cut$\leftarrow$ number of inter-cluster edges in $\mathcal{E}$;
  % Update partition with the minimal cut value
  if $Cut < Cut_{min}$ **then**
    $Cut_{min} \leftarrow Cut$; $CC_{min} \leftarrow$cutoff; $P \leftarrow \{\mathcal{C}(n)\}$

Supplementary Figure 1. Main algorithm for the clustering based on minimal cut to find pseudo-cliques. The **Split** step is detailed in Figure 2. The Merge step is not detailed as it is more trivial. Procedure sort_deg sort nodes in decreasing value of deg, then of CC. Procedure sort_degc sort nodes in decreasing value of deg, then of CC for the representative of the cluster.

1 **Algorithm:** Split

Data: Graph $\mathcal{G} = (\mathcal{N}, \mathcal{E})$.
$\mathcal{C}_i$ and $\mathcal{C}_j$ two clusters of nodes $\subset \mathcal{N}$ with a non null intersection
$\mathcal{I}_k = \mathcal{C}_i \cap \mathcal{C}_j$.
Cl is the set of clusters.

2 $cut_{\mathcal{C}_i} \leftarrow \{e_{lm} : n_l \in \mathcal{C}_i \setminus \mathcal{I}_k, n_m \in I_k, e_{lm} \in \mathcal{E}\}$;
3 $cut_{\mathcal{C}_j} \leftarrow \{e_{lm} : n_l \in \mathcal{C}_j \setminus \mathcal{I}_k, n_m \in I_k, e_{lm} \in \mathcal{E}\}$;
4 **switch $\mathbf{cut}_{\mathcal{C}_i}$ do**
5     $>$ $\mathbf{cut}_{\mathcal{C}_j}$: Remove nodes of $\mathcal{I}_k$ from C_j;
6   **if** $\mathcal{C}_j$ *is not connected anymore* **then**
7   Split($\mathcal{C}_j$) using steps 11, 12, and 13 of main algorithm[1];
8   Append new clusters in Cl;
9     $<$ $\mathbf{cut}_{\mathcal{C}_j}$:
10   Remove nodes of $\mathcal{I}_k$ from $\mathcal{C}_i$;
11   **if** $\mathcal{C}_i$ *is not connected anymore* **then**
12   Split($\mathcal{C}_i$) using steps 11, 12, and 13 of main algorithm[1];
13   Append new clusters in Cl;
14     $==$ $\mathbf{cut}_{\mathcal{C}_j}$:
15   Compute $\Delta CC_{\mathcal{C}_i,\mathcal{C}_i \setminus \mathcal{I}}$ and $\Delta CC_{\mathcal{C}_j,\mathcal{C}_j \setminus \mathcal{I}}$ (equation 4 in main text);
16   **if** $\Delta CC_{\mathcal{C}_i,\mathcal{C}_i \setminus \mathcal{I}} \leq \Delta CC_{\mathcal{C}_j,\mathcal{C}_j \setminus \mathcal{I}}$ **then**
17   Do steps 5 to 8.
18   **else**
19   Do steps 10 to 13.

Supplementary Figure 2. Node removal from one of two intersecting sets. This procedure chooses the set to shrink by keeping the minimal cut between the two. In case of ties, this procedure attributes the nodes of the intersection to the set that has the greatest gain or the lowest loss of connectivity when keeping the nodes of the intersection.

Implementation choices for scalability

Approximated minimal cut The most costly phase relies on the treatment of the largest connected components. In large connected components, many clustering coefficients values are very close. Introducing a rounding factor in when computing the *ClCo* results in a neat decrease of the number of different values observed, and thus restrains drastically the number of iterations necessary for the main loop. As a consequence, the optimization only computes an upper bound of the minimal cut.

Graph pre-processing We chose to disconnect the *articulation points* of the graph to remove nodes that can be targeted as potential bridges between two correct clusters. These are nodes whose removal increases the number of connected components in the graph. Such nodes can be spotted as problematic as we do not expect a single read to be the only link between many others. Their detection can be done with a DFS in time complexity of $\mathcal{O}(\mathcal{N} + \mathcal{E})$ for the whole graph.

Additional results

Results on toy instances

Fortunato et al. proposed to test the resolution limit of community detection on a ring of 30 cliques of 5 nodes interconnected through single links. We extend this example by proposing two toy examples that represent simplified instances in comparison to real data. However, they provide a clear illustration of the fact that our approach has the desired behavior as a community detection method, i.e. it detects each clique or quasi-clique as a distinct community, which is appropriate in our application since the set of long reads representing the isoforms of a gene are supposed to share more similarity links than between different genes and form a quasi-clique.
As the comparison with the main existing methods shows, obtaining such behaviour is not trivial.

The Louvain algorithm finds the partition in cliques at the first level of the hierarchy and builds groups of 2 cliques at the second and last level. CARNAC-LR finds the correct partition in cliques without its articulation node filter. As this instance was easy to retrieve, we slightly complicated the initial example: we made the cliques bigger (size 7) and cliques are interconnected through two links on different nodes (see Supplementary Figure 3, left). We provide an example of the resolution achieved by Louvain's algorithm on this new problem to illustrate its difficulty (Supplementary Figure 3, right). It cannot find the partition in cliques and moreover, the cliques are not always split at the same place. Contrary to *modularity*-based approaches CARNAC-LR successfully reported the 30 expected clusters of cliques (Supplementary Figure 4).

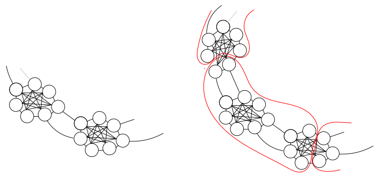

Supplementary Figure 3. On the left we provide an example to show how 7-cliques are connected in our example design. In total, a ring of 30 7-cliques is used. On the right we illustrate Louvain's result on this instance. The clusters formed by Louvain are in red, spanning several cliques.

Summary of clustering tools results

In Supplementary Table 5 we show the summary of the results of the benchmark of nucleic acid sequences clustering tools compared to our pipeline presented in *"Comparison to other nucleic sequence clustering tools"* section. It states that most of the tools simply cannot be used with long reads, those generic enough such as CD-HIT performing poorly in comparison to our method.

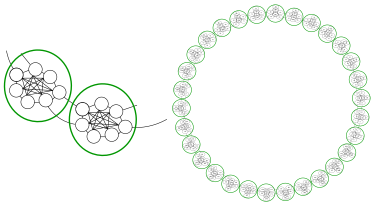

Supplementary Figure 4. CARNAC-LR result clusters on the ring of 7-cliques. A close-up on two cliques is shown on the left. Each cluster output by CARNAC-LR is represented by a green circle. It can be seen that cliques are separated from each other as our method identifies them as independent clusters.

Supplementary Table 1. Benchmark of nucleic acid sequence clustering tools (read sampling from mouse chromosome 1). Details: see caption from Supplementary Table 1.

	Recall (%)	Precision (%)	F-measure (%)	Status	time (hh:mm:ss)	Memory (Mb)
CD-HIT	11.70	**99.87**	20.95	run	03:33:2	3.11
SEED	0	0	0	run	ends immediately	-
Starcode	-	-	-	error	-	-
Rainbow	-	-	-	could not be run	-	-
Tofu	-	-	-	could no be run	-	-
CARNAC-LR + Minimap	**60.16**	98.04	**74.57**	run	00:00:16	3.99

CPM and Louvain detailed results

Louvain being a multi-level method and CPM's result being dependant on a parameter, we add other results in Supplementary Table 2 to complement the results of the main document.

Supplementary Table 2. Comparison between CARNAC-LR and the different Louvain iteration/a range of k parameters for CPM on chromosome 1 sample. Over the last value for CPM, no community is found at all.

	Recall (%)	Precision (%)	F-measure
CPM $k = 3$	63.03	87.17	73.16
CPM $k = 4$	61.99	87.02	72.41
CPM $k = 5$	52.69	88.15	65.95
CPM $k = 10$	52.69	99.37	62.60
CPM $k = 20$	37.90	99.34	54.86
CPM $k = 50$	15.10	98.97	26.21
CPM $k = 100$	4.778	99.57	9.119
CPM $k = 144$	1.488	100	2.933
Louvain lvl 1	81.01	14.71	28.89
Louvain lvl 2	82.32	13.86	27.26
CARNAC-LR	60.16	98.04	74.57

Read sampling on whole mouse dataset

We add a second way of sampling reads by randomly selecting them in the whole mouse transcriptome (14,566 mouse reads within 2342 reference clusters). We run all methods on a same input graph, that has been pre-processed using the procedure described in the main document (number of connected components before pre-processing: 2121, number of connected components after pre-processing: 3249). The input graph $\mathcal{G} = \{\mathcal{V}, \mathcal{E}\}$ has the following properties: $|\mathcal{V}| = 11,595$, $|\mathcal{E}| = 65,937$, graph clustering coefficient: 0.000065, graph average geodesic distance: 13.434290 and graph diameter: 39. A binned distribution of the degrees is presented in Supplementary Figure 5. Results are presented in

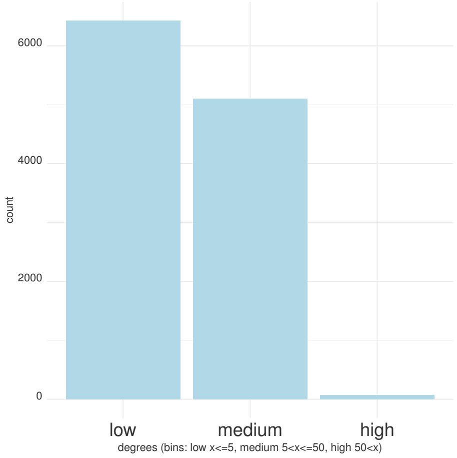

Supplementary Figure 5. Binned distribution of nodes' degrees in the input graph.

Supplementary Table 3. Again we show detailed results of Louvain and CPM

Supplementary Table 3. Comparison with state of the art methods. The benchmark was realized on a 10K reads dataset from the mouse brain transcriptome. CPM3 denotes the CPM algorithm using $k = 3$. Column "clusters" shows the number of clusters of size > 1 in the output.

	Recall (%)	Precision (%)	F-measure (%)	Jaccard index	#clusters
Transitive closure	52.95	83.93	64.932	E^{-1}	2035
Modularity	30.15	19.34	23.57	E^{-1}	2035
CPM3	45.20	82.50	58.40	E^{-1}	1149
Louvain	**64.77**	17.96	34.95	E^{-2}	738
CARNAC-LR	52.31	**88.00**	**65.62**	$\mathbf{E}^{-1}$	2052

on this sampling in Supplementary Table 4.

We present a comparison to sequence clustering tools in Supplementary Table 5.

It can be seen that for both comparisons, the result conclusions remain unchanged from those of the main document, where CARNAC-LR performs globally better on a real instance dataset.

Supplementary Table 4. Comparison between CARNAC-LR and the different Louvain iteration/a range of k parameters for CPM on chromosome 1 sample. Over the last value for CPM, no community is found at all.

	Recall (%)	Precision (%)	F-measure
CPM $k = 3$	45.20	82.50	58.40
CPM $k = 4$	39.65	81.93	53.34
CPM $k = 5$	34.48	82.03	48.55
CPM $k = 10$	21.22	80.32	33.57
CPM $k = 20$	10.89	77.48	19.10
CPM $k = 50$	1.456	100	28.70
CPM $k = 74$	0.515	100	1.025
Louvain lvl 1	81.01	14.71	28.89
Louvain lvl 2	82.32	13.86	27.26
CARNAC-LR	52.32	88.00	65.62

Supplementary Table 5. Benchmark of nucleic acid sequence clustering tools (read sampling from whole mouse genome). Each tool embeds its own strategy to compute similarity between sequences, our pipeline integrates Minimap. The three first columns present the same metrics than in previous benchmark. The *status* column indicates whether the tool could be used on ONT reads. Wallclock time and peak memory are reported in the last columns. Starcode finished with error message "does not work with sequences longer than max sequence length exceeded (1023)". The manual increase of this value in the code has led to excessive memory requirements. SEED returned an empty output. Rainbow could not be run because it explicitly asks for paired reads typical from RAD-seq short reads sequencing. Tofu pipeline specifically involves specificity of PacBio Isoseq sequences that cannot be replaced with ONT reads.

	Recall (%)	Precision (%)	F-measure (%)	Status	time (hh:mm:ss)	Memory (Mb)
CD-HIT	26.60	**99.27**	41.96	run	03:06:5	2.47
SEED	0	0	0	run	ends immediately	-
Starcode	-	-	-	error	-	-
Rainbow	-	-	-	could not be run	-	-
Tofu	-	-	-	could no be run	-	-
Minimap+ CARNAC-LR	**52.32**	88.00	**65.62**	run	00:00:13	4.00

Scalability

In Supplementary Figure 7 we plot wallclock runtimes of CARNAC-LR on different sizes of datasets samples from real data. For 10K, the graph characteristics were (N=4,340, E=125,172, number of connected components=403 , biggest connected component=194 nodes), for 100K (N=43,588, E=435,530, number of connected components=3,970 , biggest connected components=6,010 nodes) and 1M (N=439,510, E=15,043,753, number of connected components=38,506, biggest connected component=94,099 nodes) We show a roughly linear time consumption in the size of the input in the experiment. In Supplementary Fig-

ure 6 we show the gain in threading CARNAC-LR. The longest computation time is dedicated to the biggest connected component (94,099 nodes) that takes one thread. It can be seen that the memory footprint is not impacted a lot by the use of several threads.

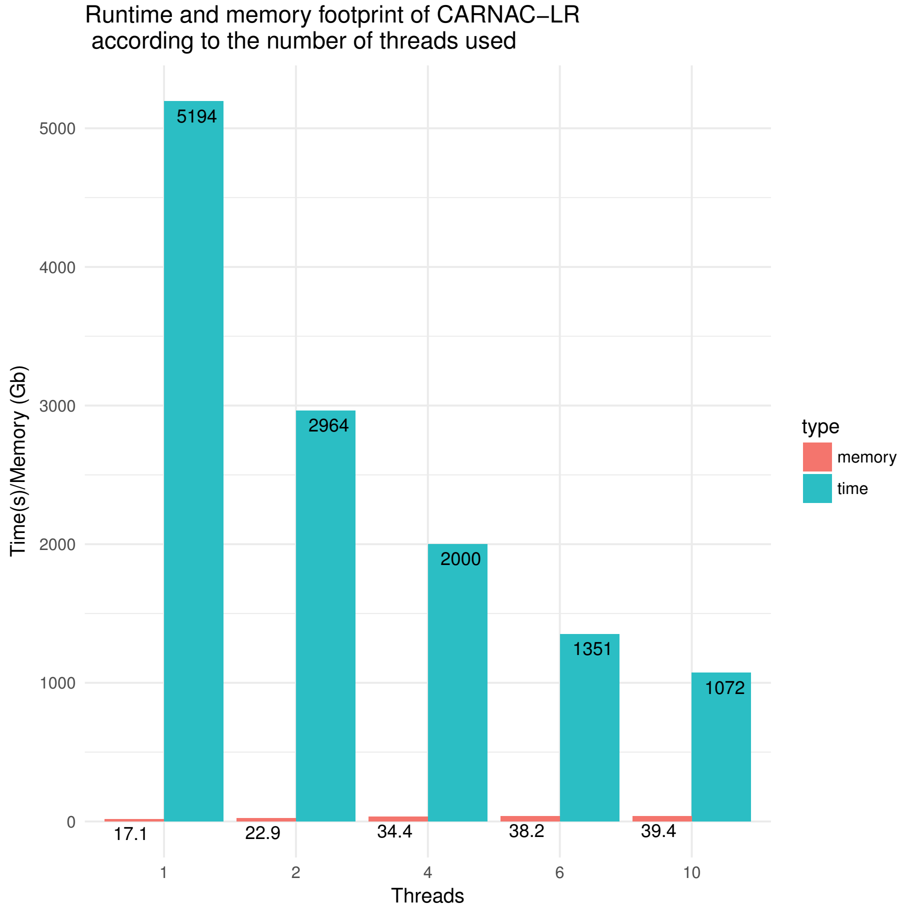

Supplementary Figure 6. Comparison of time and memory consumption and gain in throughput of CARNAC-LR on mouse dataset (1M reads) when single or multi threaded.

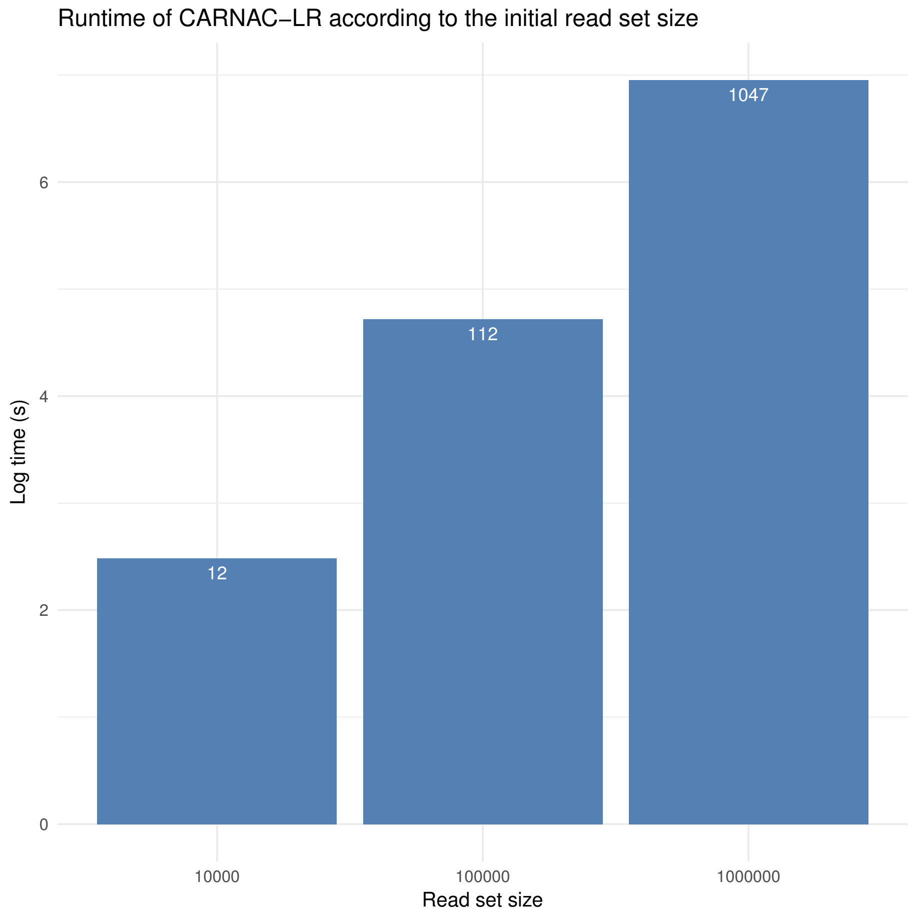

Supplementary Figure 7. Wallclock time for different sizes of datasets (10K, 100K and 1M reads) of mouse transcriptome reads (40 threads.) Time scale is log, real wallclock time values in seconds are annotated in bars.
